# Human Gamma Delta T Regulatory Cells in Cancer: Fact or Fiction?

**DOI:** 10.3389/fimmu.2014.00598

**Published:** 2014-11-20

**Authors:** Daniela Wesch, Christian Peters, Gabrielle Melanie Siegers

**Affiliations:** ^1^Institute of Immunology, Christian-Albrechts University of Kiel, Kiel, Germany; ^2^Department of Oncology, University of Alberta, Edmonton, AB, Canada

**Keywords:** gamma delta T cells, cancer immunotherapy, regulatory T cells, human cancer, gamma delta T cell functional plasticity

## Abstract

While gamma delta T cell (γδTc) anticancer immunotherapies are being developed, recent reports suggest a regulatory role for γδTc tumor-infiltrating lymphocytes. This mini-review surveys available evidence, determines strengths and weaknesses thereof and suggest directions for further exploration. We focus on human γδTc, as mouse and human γδTc repertoires differ. Regulatory γδTc are defined and compared to conventional Tregs and their roles in health and disease (focusing in on cancer) are discussed. We contrast the suggested regulatory roles for γδTc in breast and colorectal cancer with their cytotoxic capabilities in other malignancies, emphasizing the context dependence of γδTc functional plasticity. Since γδTc can be induced to exhibit regulatory properties (in some cases reversible), we carefully scrutinize experimental procedures in published reports. As γδTc garner increasing interest for their therapeutic potential, it is critical that we appreciate the full extent of their role(s) and interactions with other cell types in both the circulation and the tumor microenvironment. A comprehensive understanding will enable manipulation of γδTc to improve anti-tumor efficacy and patient outcomes.

## Introduction

While those of us in the immunotherapy world tend to focus on the anti-infection and anti-tumor properties of γδTc, we are now beginning to appreciate that, under certain conditions, these remarkable cells can inhibit or suppress the maturation and/or activation of immune cells around them, leading to beneficial or potentially pathological consequences.

**Figure 1:**
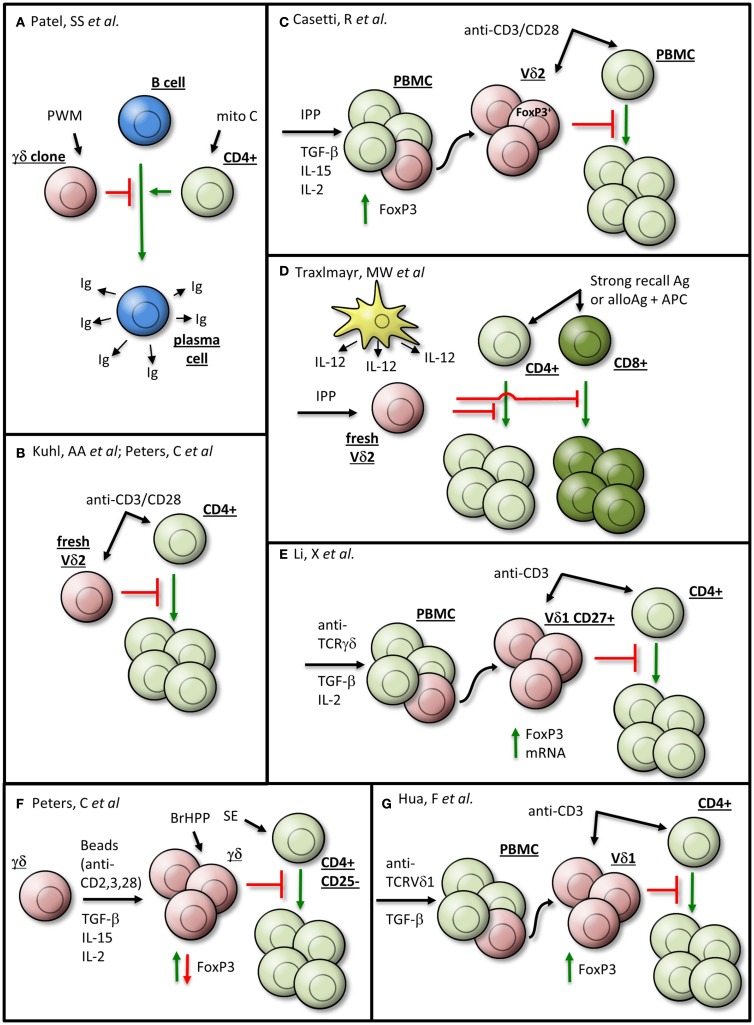
**γδTc exhibiting regulatory properties may be generated *in vitro* by various means**. Details are given in the text and the indicated references. The γδTc are depicted in red, αβTc in green, B cells in blue, dendritic cells in yellow, and senescent cells in gray. Ag, antigen; APC, antigen-presenting cell; BrHPP, bromohydrin pyrophosphate; fresh, freshly isolated; Ig, immunoglobulin; IPP, isopentenyl pyrophosphate; Mito C, mitomycin C; PBMC, peripheral blood mononuclear cell; PWM, pokeweed mitogen; SE, *Staphylococcus aureus* enterotoxins. **(A)** Patel et al., **(B)** Kuhl et al., Peters et al., **(C)** Casetti et al., **(D)** Traxlmayr et al., **(E)** Li et al., **(F)** Peters et al., and **(G)** Hua et al.

A suppressor function of human γδTc was first described in 1989 by Patel and colleagues; upon *in vitro* stimulation with pokeweed mitogen, most γδTc clones could suppress the generation of Immunoglobulin(Ig)-secreting B cells by CD4^+^ T helper cells treated with mitomycin C (Figure 1A) (1). Since then, regulatory roles for γδTc have been described in several contexts. Both Vδ1 and Vδ2 T cell subsets (Vδ1Tc and Vδ2Tc, respectively) may exhibit regulatory properties, albeit in different settings.

Human peripheral blood-derived γδTc displaying regulatory properties are phenotypically different from conventional regulatory CD4^+^ αβ T cells (Treg). In contrast to Treg, freshly isolated γδTc express only low levels of CD25 and cytotoxic T lymphocyte-associated antigen (CTLA)-4, and do not express the transcription factor forkhead box P3 (FoxP3) (2–4). Similar to conventional αβ T cells (αβTc), CD25 is up-regulated on γδTc after initial phytohemagglutinin (PHA) or anti-γδ TCR monoclonal antibody (mAb) stimulation (5). Additionally, CD25 is also up-regulated on Vδ2Tc after stimulation with pyrophosphates (phosphorylated antigens), which are intermediates of the isoprenoid pathway and induce selective expansion of Vδ2Tc within peripheral blood mononuclear cells (PBMC) 7–10 days after initial stimulation (6, 7). Furthermore, FoxP3 expression can be detected with PCH101 mAb but not with the more Treg-specific 259D mAb, in γδTc as well as in Treg-depleted αβTc after activation (4). FoxP3 expression as identified by PCH101 mAb does not correlate with suppressive function (8, 9). In addition, the transcription factor Helios, which is highly expressed by Treg, is constitutively expressed in roughly one-third of freshly isolated γδTc (4). While Helios seems to be involved in the differentiation of (regulatory) γδTc, it is not a specific marker for suppressive γδTc (4, 10). Thus, while freshly isolated γδTc do not express characteristic Treg markers, the literature provides evidence that γδTc may nevertheless exhibit regulatory activity, which will be further described below.

## Regulatory Roles for γδTc in Non-Cancer Contexts

Before focusing in on the potential regulatory role of γδTc in cancer, it is worthwhile to consider some other contexts in which these cells have displayed suppressive properties. For a more comprehensive description of regulatory roles of γδTc outside of cancer, we recommend a recent review (10).

Immunosuppression via γδTc plays a protective role in several contexts. For example, in pregnancy, decidual γδTc contribute to an immunosuppressive milieu enabling successful implantation and protecting the growing fetus from attack by the mother’s immune system (11–14). In celiac disease, patients on a gluten-free diet have enhanced suppressor intestinal intraepithelial γδTc that protect the small intestine from attack by CD8^+^ TCRαβ^+^ intraepithelial lymphocytes (IEL) via secretion of transforming growth factor-beta one (TGF-β1); patients with active disease have lower frequencies of these suppressor γδTc IEL (15). Lower peripheral blood γδTc numbers, more specifically a decreased proportion of central memory γδTc, are correlated with systemic lupus erythematosus pathogenesis, suggesting a protective role for regulatory γδTc in this autoimmune disease as well (16). Of note, Vδ1Tc/Vδ2Tc subset ratios are inverted in patients compared to healthy controls (i.e., Vδ1Tc predominate in blood) (16). Similarly, a higher Vδ1Tc/Vδ2Tc ratio may contribute to the achievement of operational tolerance in pediatric liver transplant recipients (17).

## How to Make Regulatory γδTc

So far, it is unknown whether specific subsets, e.g., CD27^+^ Helios-expressing γδTc, are innately suppressive or whether their broad range of functional plasticity enables suppressive activity under certain stimulatory conditions (Figure 1). An observation common to all studies on suppressive Vδ2Tc is that they realize their immunosuppressive potential only in the presence of antigen-presenting cells (APC) or after co-stimulation with anti-CD28 mAb (Figures 1B,C) (2–4). CD28 and CTLA-4 are critical regulators of immunosuppressive T cells, whereby CD28 plays a dual role in both the generation and the termination of an immune response (18).

Freshly isolated isopentenyl pyrophosphate (IPP)-stimulated Vδ2Tc can inhibit the proliferation of CD4^+^ and CD8^+^ αβTc in response to strong recall antigens such as Tetanus toxoid, superantigens such as S*taphylococcus aureus* enterotoxins (SE) or alloantigens in the presence of APCs (Figure 1D) (19). However, the authors could not completely rule out low frequency activation of αβTc by antigen-specific (e.g., Tetanus toxoid) stimulation. Nevertheless, peripheral blood Vδ2Tc also suppress proliferation of co-cultured CD4^+^ αβTc after polyclonal stimulation by anti-CD3/CD28 mAb, which simultaneously activates αβTc (Figure 1B) (3, 4). All in all, the presence and strength of a co-stimulatory APC-signal seem to play an important role in the induction of Vδ2Tc suppressive capacity (4).

While TGF-β1 alone does not induce the generation of regulatory Vδ2Tc, this switch can occur in the presence of additional cytokines (Figures 1B,C,E,F) (2, 4, 9, 16). Up to 30% of Vδ2Tc within IPP-stimulated PBMC cultivated in the presence of TGF-β1 and interleukin (IL)-15 expressed FoxP3 (clone 259D); after subsequent cell sorting, these FoxP3^+^ enriched Vδ2Tc suppressed the proliferation of anti-CD3/CD28 mAb-simulated PBMC (Figure 1C) (2). Peters and colleagues have since demonstrated that the observed FoxP3 expression was transient, with a steady increase in FoxP3 over 8 days of cell culture followed by a decrease to nearly undetectable protein levels after 16 days (4).

In contrast to the work of Casetti and colleagues, in the study of Peters et al. TGF-β1 and IL-15 did not induce regulatory functions in bromohydrin pyrophosphate (BrHPP)-expanded γδTc. Only anti-CD3/CD28 mAb-stimulated γδTc expanded in the presence of TGF-β1 and IL-15 were able to suppress the proliferation of αβTc induced by a mixture of SE (Figure 1F) (4). The observed suppressive activity was not dependent on FoxP3 expression but was rather dependent on the presence of initial CD28-co-stimulation. The discrepancy between these two studies might be explained by differences in γδTc expansion as well as stimulatory conditions in the suppression assays. Casetti et al. used IPP-stimulated PBMC from which Vδ2Tc were sorted after expansion, whereas Peters et al. expanded magnetically isolated, highly purified γδTc (20). In their suppression assay, Casetti et al. analyzed the Vδ2Tc suppression of PBMC stimulated by anti-CD3/CD28 mAb, which could potentially activate other suppressive T cell subsets within the PBMC. In contrast, Peters and colleagues used CD25-depleted CD4^+^ T cells as responder cells, which were stimulated by a mixture of SE and BrHPP-restimulation for the co-cultured γδTc. Common to both studies is a correlation between CD28-co-stimulation (although at different time points) and the suppressive effect. This suggests that CD28 signaling in γδTc-mediated suppression should be examined in more detail.

While FoxP3 and γδTc regulatory activity are not strictly connected, it is worthwhile to note that FoxP3 expression can be induced in both Vδ1Tc and Vδ2Tc subsets. Similar to Vδ2Tc, FoxP3 was prominently induced in Vδ1Tc in the presence of TGF-β1 and additional cytokines such as IL-2 after stimulating PBMC with anti-γδTCR for 10 days (16). Additionally, there was an increased expression of both TGF-β1 and its receptor (CD105) on Vδ1Tc compared to Vδ2Tc; upon activation, TGF-β1 decreased and CD105 increased on Vδ1Tc. The authors assumed a regulatory role for the Vδ1 CD45^−^CD27^+^ γδTc subset due to its increased FoxP3 expression. While they demonstrated inhibition of CD4^+^ T cell proliferation by CD27^+^ Vδ1Tc, the authors unfortunately did not directly compare the suppressive activity of CD27^+^ versus CD27^−^ Vδ1Tc (Figure 1E) (16). In this context, the analysis of FoxP3 expression in purified Vδ2Tc versus Vδ1Tc under different culture conditions would be interesting.

Finally, Hua and colleagues induced regulatory Vδ1Tc *in vitro*, upon stimulation of PBMC with plate-bound anti-TCRVδ1 mAb, that expressed FoxP3 (identified by mAb clone 259D/C7) and suppressed CD4^+^ T cell proliferation (Figure 1G) (21). The authors suggested that Vδ1Tc FoxP3 expression was sustained by a positive feedback loop instigated by Vδ1Tc producing TGF-β1; in addition, Vδ1Tc secreted IL-10 (21).

In summary, it is difficult to compare these studies, as their inherent differences in experimental design (cell source/subset/milieu/stimuli) are further confounded by the lack of a defined regulatory γδTc marker. However, it is clear that γδTc can be induced to exhibit regulatory properties.

## How Do γδTc Suppress Other Cells?

There are, however, some controversial data regarding mechanism(s) of suppression by γδTc. Kühl and colleagues assumed mediation by the immunosuppressive cytokines TGF-β1 and IL-10, which were secreted by γδTc after anti-CD3/CD28 mAb stimulation (Figure 1B). After 48 h stimulation, γδTc secreted significantly more TGF-β1 than conventional CD4^+^CD25^+^ Tregs (3). Unfortunately, their ELISAs did not distinguish between TGF-β1 secretion by Vδ1Tc and Vδ2Tc; however, higher TGF-β1 mRNA levels after 3 day Concanavalin A treatment would suggest that Vδ1Tc have a greater suppressive capacity than Vδ2Tc or αβTc (3).

**Figure 2:**
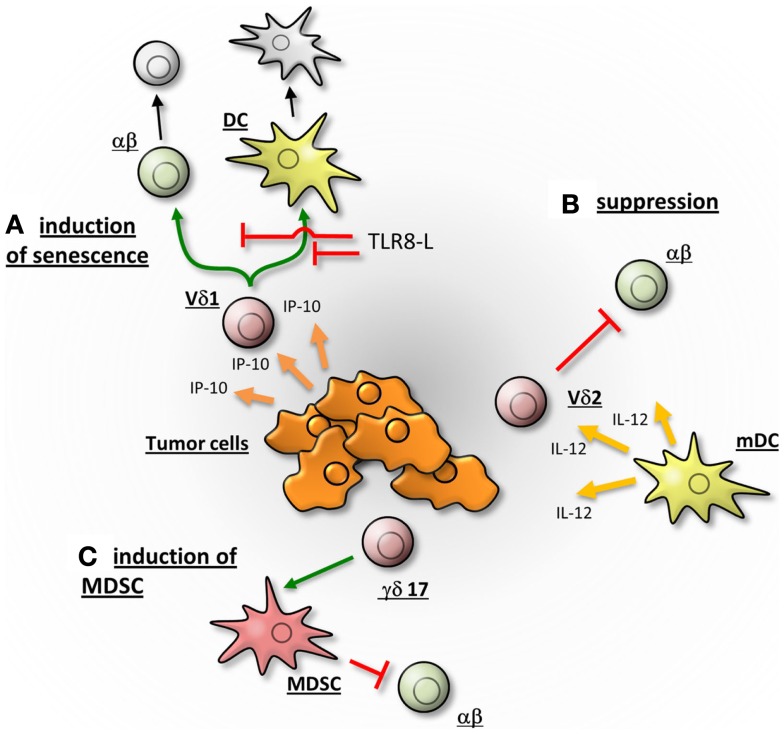
**γδTc may promote the tumor growth by different mechanisms**. **(A)** According to Peng and colleagues, Vδ1Tc are attracted by IP-10 and migrate into the breast-tumor microenvironment. There they induce the senescence of αβTc and dendritic cells (DC), thereby suppressing an immune response. The induction of senescence can be abrogated in a TLR8 dependent manner. **(B)** Vδ2Tc, activated by IL-12 secreting DC, suppress αβ T cells and thereby potentially hamper an anti-tumor response of αβTc. **(C)** γδ17 T cells may support tumor progression by the promotion of angiogenesis and the induction of myeloid-derived suppressor cells (MDSC), which in turn suppress an αβTc immune response. The γδTc are depicted in red, αβTc in green and senescent cells in gray.

Peters and co-workers demonstrated that co-culture with responder cells (CD25-depleted CD4^+^ αβTc) induced the upregulation of CD80 and CD86 as well as programed death-ligand (PDL)-1 on stimulated Vδ2Tc, which could then interact with CTLA-4 or PD-1 on responder cells, leading to their suppression (4). Furthermore, transwell experiments suggested cell-contact-dependence, as this process was inhibited by mAb disrupting CD86:CTLA-4 or PDL-1:PD-1 interactions between anti-CD3/CD28 mAb-activated Vδ2Tc and activated αβTc (4). Interestingly, the immunosuppressive capacity of Vδ2Tc was abrogated by Toll-like-receptor (TLR) 2 ligands as well as by activating αβTc with a mixture of five *SE* (in contrast to the publication of Traxlmayr where only one SE was applied), which both induce a strong Th1-response [(4, 19); Peters and Wesch, unpublished data]. Abrogated suppression correlated with increased phosphorylation of Akt and NFκB in αβTc and down-regulation of inhibitory molecules such as PD-1 and CTLA-4 (4). Similarly, Peng and colleagues found that the regulatory γδTc phenotype could be reversed through administration of TLR8 ligand Poly-G (Figure 2A) (22, 23). Only ligands to TLR8 (and not TLRs 2, 3, 4, 5, 7, or 9) blocked induction of senescence observed in T cells responding to suppression via regulatory γδTc (Figure 2A) (23). These observations exemplify the functional plasticity of γδTc that are influenced by the nature of a stimulus and the surrounding cytokine milieu.

An important question is how TGF-β1 induction of conventional Tregs compares to that of regulatory γδTc. Li and colleagues provided evidence that TGF-β1-stimulated CD25^+^CD27^+^ Vδ1Tc exert a suppressive effect on naïve CD4^+^ T cells similar to classical Tregs, and that this mechanism was cell-cell contact dependent (16) as described for Vδ2Tc above (4).

## Reports of Regulatory γδTc in Cancer

While several studies have proven the cytotoxic capabilities of circulating γδTc and *in vitro-*expanded γδTc derived thereof [reviewed in Ref. (24–29)], γδTc tumor-infiltrating lymphocytes (TIL) may have very different functional properties (Figure 2). The tumor microenvironment (TME) is characterized in part by the presence of immunosuppressive cytokines such as TGF-β1 and IL-10 that prevent immune attack against the growing malignancy. Thus, one might assume that this environment would support the generation of regulatory γδTc; however, to date only very few reports support this assumption.

In a study looking at T cells from blood and TIL from lung cancer patients, freshly isolated γδTc only slightly expressed FoxP3 compared to CD4^+^ T cell TIL, of which almost half were positive for this regulatory marker (9). Blood-derived γδTc stimulated with anti-γδ TCR mAb for 14 days *in vitro* expressed only low levels of FoxP3, regardless of whether from healthy donors or lung cancer patients. Somewhat higher FoxP3 expression was evident in TIL-derived γδTc from renal cell carcinoma, chromaffin tumor and especially gastric cancer, with the latter comprising 21% of expanded γδTc in the given example. Furthermore, Vδ1Tc FoxP3 expression was greater than that of Vδ2Tc in expanded TILs from renal cell carcinoma (9). However, the authors admitted the inherent drawback that induction was detected by FoxP3 mAb clone PCH101, which is sensitive to cell activation (unlike clone 259D) (9); this has since been further corroborated (4, 9). While researchers attempting to characterize γδTc TIL in various cancer contexts have investigated expression of FoxP3, they have failed to consistently correlate its expression to regulatory function. Thus, we conclude that FoxP3 expression is an inappropriate proxy for γδTc regulatory potential and thus should be regarded with caution.

After vaccination, increased *in vitro* proliferation of Vδ2Tc from bone and connective tissue sarcoma patients undergoing immune therapy with autologous IL-12 secreting dendritic cells (DC; initially treated with tumor-derived soluble antigen plus lipopolysaccharide (LPS) and interferon (IFN)-γ: Trivax) was observed. Gene expression profiling experiments indicated an over-expression of hydroxy-methylglutaryl-CoA reductase (HMGR) in LPS/IFN-γ-stimulated- compared to unstimulated DC. HMGR is the rate-limiting enzyme of the mevalonate pathway that enhanced IPP levels leading to Vδ2Tc activation. Further *in vitro* studies revealed a suppressive potential of Vδ2Tc expanded by phosphoantigens (IPP) in the presence of IL-12 secreting DC (Figure 2B) (19).

While *in vitro-*expanded peripheral blood-derived γδTc kill human breast cancer cells (30) and *in vivo* methods to expand γδTc targeting breast cancer have already been employed in clinical trials (31, 32), a recent study of TIL in human breast tumors deemed γδTc the most significant predictor of negative outcome (33). γδTc frequency was correlated with negative factors such as advanced tumor stage, positive lymph node status, and human epidermal growth factor receptor 2 (HER2) expression. Exhaustive statistical analysis correlated γδTc with FoxP3^+^ cells (identified with clone 236A/E7) and inversely with CD8^+^ cytotoxic Tc, suggesting a negative role for γδTc (33). However, double staining of γδTc and FoxP3 was not done, leaving the identity of FoxP3^+^ cells ambiguous, and there was no indication as to whether staining was performed on serial sections. Furthermore, γδTc subsets were not specified, likely due to a dearth of subset-specific antibodies suitable for their detection via immunohistochemistry (33). Finally, while γδTc frequency in breast tumors may prove to be a valuable prognostic marker, their role in disease pathogenesis was not determined.

This same group, however, had previously suggested regulatory properties for Vδ1Tc TIL in breast tumors (22). γδTc TIL were extracted from a digested human breast tumor, expanded *in vitro* for 1 week in 1000 IU/ml IL-2, after which bulk TILs were maintained at 50 IU/ml IL-2. Tumor-reactive clones were then generated and both the bulk population and selected clones derived thereof suppressed naïve T cell proliferation, IL-2 secretion, and DC maturation (22). This may not reflect the case *in situ*. While this study proves that Vδ1Tc can assume a regulatory phenotype, several caveats demand attention:

Firstly, the subset prevalence of γδTc in the original tumor was not reported and thus (regulatory) Vδ2Tc may have comprised the majority of tumor-derived cell suspensions at the outset but may have been subsequently eliminated by high levels of IL-2 in the culturing process, since Vδ2Tc are known to be susceptible to activation-induced cell death (34–36). Broad ranges of Vδ1Tc levels were only determined after culturing, while Vδ2Tc percentages were not reported (22). In a follow-up paper, recruitment of γδTc with a regulatory phenotype was linked to high levels of IFN-γ inducible protein 10 (IP-10) in the TME (Figure 2A); however, Vδ1Tc and Vδ2Tc were unfortunately not distinguished (37). Secondly, the high level of IL-2 used to culture TILs may in itself have supported expansion of a regulatory phenotype not truly reflective of the original functional orientation of these cells. Thirdly, most experiments were carried out with one cell line and clones derived from a single tumor, thus cannot represent a universal truth. It is also not clear whether the same Vδ1Tc lines were used in subsequent publications. While valuable insight into the plasticity and regulatory potential of Vδ1Tc can be gleaned from these studies, further investigation of γδTc TIL *in situ* are required to substantiate claims of regulatory function contributing to poor patient prognosis.

While breast-tumor TIL-derived Vδ1Tc can exhibit regulatory properties *in vitro*, Vδ1Tc TIL from other cancers have been reported to be cytotoxic (38, 39). Polyclonal γδTc TIL lines kill melanoma cell lines, and secrete tumor necrosis factor alpha (TNFα) and IFN-γ (38). This functional diversity could well be context-dependent or perhaps, as Donia and colleagues suggest, clones with various Vγ pairings are differentially activated (39). It is also possible that these cytotoxic γδTc TIL are simultaneously capable of as-of-yet unnoticed regulatory functions.

Finally, an indirect regulatory role for γδTc has been reported in colorectal cancer (CRC), whereby IL-17 secreting γδTc (γδ17) in the TME may attract and help support immunosuppressive myeloid-derived suppressor cells (MDSC) (Figure 2C). *In vitro* experiments showed that activated inflammatory DC secrete IL-23 facilitating the generation of γδ17. DC activation is thought to be caused by release of bacterial products through the compromised epithelial barrier characterizing CRC. Of note, γδ17 isolated from CRC tumors were predominantly Vδ1Tc, secreted higher levels of IL-17 compared to normal tissue controls and did not secrete IL-4, IL-22 or immunosuppressive IL-10 (40).

## Avenues to Explore

If γδTc TIL are indeed regulatory, it is crucial to determine whether they are inherently so or whether factors in the TME induce this function. If the former is true, then presumably infusion of large numbers of cytotoxic γδTc into patients should cause no safety concern (with respect to the further promotion of tumor growth). However, if the latter is true, we need to find a way to target the TME to prevent a potentially detrimental shift to a regulatory phenotype. Better models mimicking the human TME could help us address this question.

Since γδTc can be induced to realize regulatory potential in various ways, including those involving cytokines typically present in the TME, some degree of regulatory function is plausible. However, so far the evidence is scant, limited to *in vitro* experiments with *ex vivo* expanded γδTc. Admittedly, there is an inherent difficulty in assessing the regulatory capacity of γδTc TIL *in situ*, as they are only present in relatively low abundance. Ye and colleagues attempted to address this by performing experiments with freshly purified γδTc from tumor tissues; however, depending on the nature of the antibodies used for purification, γδTc function may already have been altered (23). Finally, as discussed above, assessment using markers such as FoxP3 should be considered carefully because not every mAb clone detecting FoxP3 expression denotes regulatory function.

## Concluding Remarks

Clearly, a more reliable panel of markers or epigenetic signature correlated to the regulatory phenotype of γδTc will be required for us to assess their true function(s) *in situ*. Furthermore, a clear distinction should be made between Vδ1Tc and Vδ2Tc, which may differ dramatically in terms of plasticity and function depending on their localization and exposure to various stimuli/cytokine milieus. γδTc can be both cytotoxic and/or regulatory; therein lies their incredible therapeutic potential in the contexts of autoimmune diseases and cancer. A fuller understanding of these processes should enable us to manipulate γδTc plasticity to ensure optimal efficacy and ultimately improve patient outcomes.

## Conflict of Interest Statement

The authors declare that the research was conducted in the absence of any commercial or financial relationships that could be construed as a potential conflict of interest.
